# Internal Structures of the Globus Pallidus in Patients with Parkinson’s Disease: Evaluation with Phase Difference-enhanced Imaging

**DOI:** 10.2463/mrms.mp.2015-0091

**Published:** 2016-12-22

**Authors:** Satoru Ide, Shingo Kakeda, Tetsuya Yoneda, Junji Moriya, Keita Watanabe, Atsushi Ogasawara, Koichiro Futatsuya, Norihiro Ohnari, Toru Sato, Yasuhiro Hiai, Atsuji Matsuyama, Hitoshi Fujiwara, Masanori Hisaoka, Yukunori Korogi

**Affiliations:** 1 Department of Radiology, University of Occupational and Environmental Health, School of Medicine, 1-1 Iseigaoka, Yahatanishi-ku, Kitakyushu, Fukuoka 807-8555, Japan; 2 Department of Medical Physics in Advanced Biomedical Sciences, Faculty of Life Sciences, Kumamoto University, Kumamoto, Japan; 3 Department of Pathology and Oncology, University of Occupational and Environmental Health, School of Medicine, Fukuoka, Japan; 4 Department of Surgical Pathology, University of Occupational and Environmental Health, School of Medicine, Fukuoka, Japan

**Keywords:** phase difference-enhanced, magnetic resonance imaging, Globus Pallidus, medial medullary lamina

## Abstract

**Purpose:**

The medial medullary lamina (MML) separates the medial globus pallidus (GPm) from the lateral. The aim of this study was to assess the changes in appearance of MML related to age using the phase difference-enhanced (PADRE) imaging and to determine whether PADRE can depict the MML in the patients with Parkinson’s disease (PD).

**Materials and Methods:**

We enrolled 20 patients with PD and 50 normal control subjects (NC). First, for the visualization of the MML in the NC, we compared the PADRE, susceptibility-weighted imaging (SWI)-like images and T_2_weighted imaging (WI) by using multiple comparison. The grading methods are as follows: grade 1; MML was not delineated, grade 2; less than half of MML was delineated, grade 3; more than half of MML was delineated and grade 4; whole MML was clearly delineated. We determined grade 3 and 4 as good depiction, delineating the GPm. Then, we evaluated patients with PD using the same method.

**Results:**

In NC, the delineation of MML was good in 84% of cases on PADRE, but only 34% of cases showed a good depiction on SWI-like images (average grading score 3.31 vs 2.11, *P* < 0.05). No MML was delineated in all cases on T_2_WI. Although younger subjects tended to show whole MML clearly, a part of MML tends to be obscured with age on PADRE. In patients with PD the depiction of MML on PADRE was also good in 90% of cases.

**Conclusion:**

The PADRE technique facilitates the depiction of the MML within globus pallidus (GP) on a broad range of age NC and patients with PD and it is superior to SWI-like images and T_2_WI.

## Introduction

Deep-brain stimulation (DBS) is a reversible stereotactic neurosurgical technique that has remarkable therapeutic benefits for otherwise treatment-resistant neurological and psychiatric disorders including dystonia, Parkinson’s disease (PD), and tremor.^1–5^ The US Food and Drug Administration approved DBS with medial Globus Pallidus (GPm) stimulation for the treatment of advanced PD in 2003. Anatomically, the GPm is surrounded by the optic tract ventrally, the lateral globus pallidus (GPl) laterally and dorsally, and the posterior limb of the internal capsule (Ci) posteromedially. A thin layer, the medial medullary lamina (MML), separates the GPm from the GPl.^6,7^ Although the detailed composition of the MML remains unknown,^8^ some portion derives from nerve fibers of the striatum.

As DBS involves the insertion of electrodes into specific target structures of the brain and subsequent electric stimulation by an implanted brain pacemaker,^9^ the accurate placement of the DBS electrodes into the target site is critical.^10^ Preoperative stereotactic magnetic resonance imaging (MRI) is used to depict the DBS target structures including the anatomical variability in its position, functional segregation, and size.^1,11^ Although stereotactic imaging data are frequently calculated pre-operatively from T_2_-weighted fast spin echo-^12,13^ or proton density weighted MRI,^14^ the MML was not conclusively visualized on these images which resulted in no obvious delineation between GPm and GPl. Recently Nölte et al.^15^ who compared quantitative T_1_- and T_2_WI, ${\text{T}}_{2}^{*}\text{WI}$ , and susceptibility-weighted imaging (SWI) reported ${\text{T}}_{2}^{*}\text{WI}$ as the most promising sequence for the delineation of the MML, although they studied only healthy young adults and one PD patient. Therefore, the optimal imaging approaches for depicting the MML have not yet been clearly established.

We developed a phase-weighted MRI technique we call “phase difference enhanced (PADRE)” imaging in which the phase difference between the target and surrounding tissue is selected to enhance the contrast of the target tissue.^16,17^ By choosing appropriate phase differences this technique can create various contrasts between tissues using single MRI data. Consequently, it can be expected to improve the depiction of the MML which differentiates the GPm from the GPl. Our objective in the current study was to assess whether high-spatial-resolution PADRE imaging can delineate the MML in wide range normal control subjects (NC), moreover in elderly patients with PD.

## Materials and Methods

### Subjects

Our institutional review board approved this retrospective study and waived informed consent. At our institution, the three dimensions (3D) multi-echo spoiled gradient echo (GRE) sequence is part of routine brain MRI for indications including: 1) screening of minor hemorrhage, 2) evaluation of vascular disease, movement disorder, or degenerative disease, and the PADRE and SWI-like images were reconstructed with the MR data. From this database between January 2009 and November 2012, the study included 20 consecutive patients with PD (11 females, 9 males; mean age 69.2 years ± 8.2, range 53–83 years). PD was diagnosed by two neurologists (K.O, with 18 years of experience in movement disorders, and S.T., with 30 years of experience in movement disorders). All patients with PD fulfilled the UK Parkinson’s Disease Brain Bank criteria for the diagnosis of idiopathic PD. The median Hoehn and Yahr (H&Y) stage^18^ of the patients with PD was 3 (range 1–3). From the same database between January 2009 and November 2012, 50 consecutive NC without a history of neurological or psychiatric diseases were also selected (35 females, 15 males, mean age 54.5 years, age range 22–83 years, age distribution 20–29 years [*n* = 6], 30–39 years [*n* = 7], 40–49 years [*n* = 8], 50–59 years [*n* = 4], 60–69 years [*n* = 13], 70–90 years [*n* = 12]). Indications for their examination included headache, anterior communicating or middle cerebral artery aneurysms, bilateral upper extremity numbness, and benign positional vertigo. The 50 NC included 20 cases that were age- and sex-matched with the PD group (12 females, eight males; mean age 69.2 years ± 8.3, range 57–83 years).

### MR imaging

All studies were performed with a 3T MR system (Signa EXCITE 3T; GE Healthcare, Milwaukee, WI) using a dedicated eight-channel phased-array coil (USA Instruments, Aurora, OH). The PADRE was performed with a 3D multi-echo spoiled GRE sequence.^19^ The imaging parameters included: axial or coronal planes covering the whole globus pallidus (GP); 11 echo times; first echo time, 4.5 msec; uniform echo time spacing, 5 msec; repetition time, 58.4 msec; flip angle, 15°; bandwidth per pixel, ± 62.5 Hz; field of view, 22 × 16.5 cm; acquisition matrices, 320 × 416; slice thickness, 1.5 mm; number of slices, 1848 and an imaging time of 7 min 1 sec. A parallel imaging method (the array spatial sensitivity encoding technique) was used with a reduction factor of 2. For the 3D multi-echo spoiled GRE sequence, we acquired 33 axial and 17 coronal-plane images of the 50 NC, and all images of patients with PD were obtained in axial-plane images. All the subjects were also imaged with T_2_WI and obtained with axial plane, and imaging parameters were as follows: echo time, 85 msec; repetition time, 4000 msec; flip angle, 90°; slice thickness, 5 mm; field of view, 22 × 22 cm; acquisition matrices, 512 × 512 and an imaging time of 3 min 20 sec.

### PADRE technique

Phase data contain a “streak” rooted in the 2-pi symmetry of the phase information (image). To use this information for a diagnosis, the phase streak should be removed from phase images. The high-pass (HP) filter technique that involves a complex division between the original Magnetic Resonance (MR) image and the low-pass (LP) filtered image can be adapted to remove these streaks. Since HP filtration was achieved throughout the study by using the homodyne method we used its kernel size as the size of the HP filter.^20–22^

The HP filtered phase image is interpreted as an image of the phase difference because the HP filter removes the effects of a static magnetic field; this produces a residual magnetic field difference. The resulting phase difference information is classified in accordance with the physical magnetic property of the tissue, namely, the susceptibility of the tissue, which depends on the magnetism of the molecules and the Lorentz effect of the tissue. Therefore, selection of a specific phase difference reflects the magnetic properties of a tissue. A major concept responsible for the power of the PADRE technique is the “phase difference selection” which enhances the magnetic properties of the target tissue. SWI, for example, selects only phase differences related to vessels, especially veins. PADRE imaging, on the other hand, classifies and selects various phase differences, Δ*θ*, to enhance different tissues, and enhances all of them on the magnitude image |*ρ*| by the enhancing function *w* (Δ*θ*). Finally, the PADRE image *ρ_PADRE_* is reconstructed as:

$$\rho_{\mathrm{PADRE}}=w(\Delta \theta )|\rho |$$

Although an arbitrary enhancing function can be used, we applied an exponential function of the phase difference because such a function can locally fit any kind of polynomial function, like the enhancing function of SWI. Therefore, PADRE imaging can also achieve the contrast of SWI by selecting the phase differences mainly arising from the paramagnetism of deoxyhemoglobin, hemosiderin, etc. Although the water molecule is the main source of MR signals, SWI is not sensitive to water-rich materials because water molecules are diamagnetic and therefore, the phase difference of water-rich materials almost always shows a value different from paramagnetism.^16^

Before evaluation, we optimized the reconstitution parameters of PADRE on the basis of our previous results.^16^ In addition, we also reconstructed SWI-like images from the MR data.

Because the 3D multi-echo spoiled GRE sequence used in this study consisted of 11 kinds of TE, we were able to create the PADRE images with different TE of 4.5, 9.4, 14.4, 19.3, 24.3, 29.2, 34.1, 39.1, 44.0, 49.0 and 53.9 msec. First, one neuroradiologist (S.K. with 21 years of experience) investigated the effects of varying the TE on the contrast of the MML, and found the best delineation of the MML was obtained at a TE of 34.1 msec. Therefore, for all PADRE evaluations, a TE of 34.1 msec was chosen.

## Qualitative Assessments

**Fig 1.**
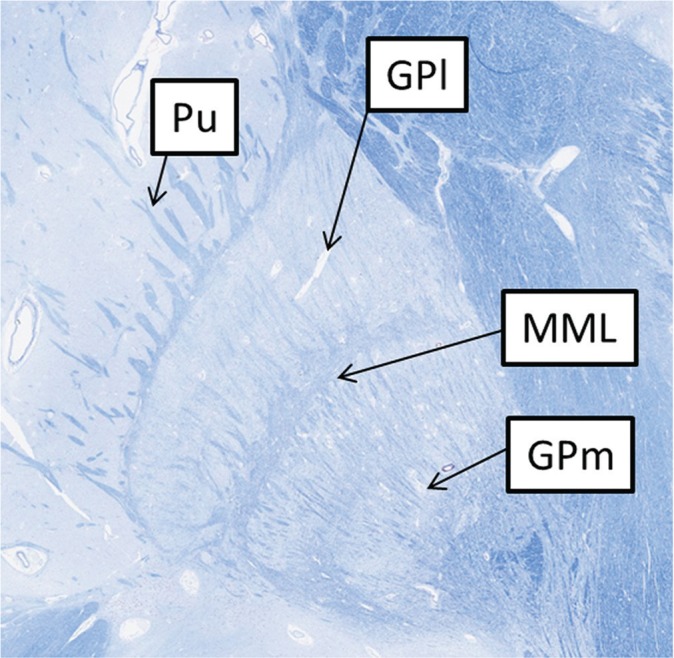
Kluver-Barrera stained coronal sections of the brain from a study subject without neurologic disease (control) at the level of the GP. A thin layer within the GP, the MML, separates the GPm from the GPl. GP, globus pallidus; MML, medial medullary lamina; GPm, medial globus pallidus; GPl, lateral globus pallidus; Pu, putamen.

**Fig 2.**
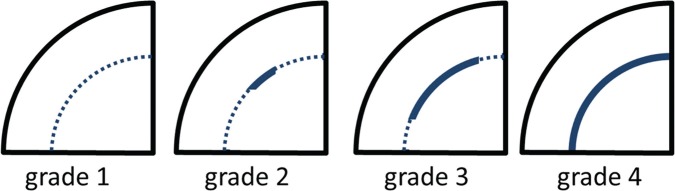
Schemas for grading the MML within GP. grade 1: MML was not delineated, grade 2: less than half of MML was delineated, grade 3: more than half of MML was delineated, grade 4: whole MML was clearly delineated. GP, globus pallidus; MML, medial medullary lamina.

Two neuroradiologists (N.O. with 30 years of experience, and J.M. with 15 years of experience) who did not take part in image manipulation independently reviewed the PADRE, SWI-like images and T_2_WI. They were cognizant of the subjects’ age and sex but blinded to clinical data. On the basis of the anatomical appearances of a cadaveric specimen stained with the Kluver-Barrera method, which enhance the myelin of tissue, (Fig. 1) and the Schaltenbrand and Wahren,^23^ two neuroradiologists classified the delineation of the MML within GP in each study subject into 4 grades (Fig. 2), where grade 1 (MML was not delineated), grade 2 (less than half of MML was delineated), grade 3 (more than half of MML was delineated) and grade 4 (whole MML was clearly delineated) using the NC. We determined grade 3 and 4 as good depiction, delineating of the GPm. We divided the NC into six age groups (20–29 years [*n* = 6], 30–39 years [*n* = 7], 40–49 years [*n* = 8], 50–59 years [*n* = 4], 60–69 years [*n* = 13], and 70–89 years [*n* = 12]), and evaluated an age-related change of the delineation of the MML. Then, in patients with PD and age-matched NC subjects, we evaluated the depiction of the MML using the same methods. And we also evaluated the relationship between the depiction of the MML in patients with PD and H&Y stages. Two neuroradiologists also evaluated the PADRE and SWI-like images for the delineation of the Ci and recorded Ci as “visible” or “not visible”. Both neuroradiologists solved all disagreements by consensus reading of images. Their consensus grading scores were used in the analyses; the PADRE was compared with the SWI-like images and T_2_WI. For these comparisons, we divided the patients with PD and age-matched NC subjects into two age groups (50–69 years [*n* = 10] and 70–89 years [*n* = 10] for PD, and 50–69 years [*n* = 11] and 70–89 years [*n* = 9] for age-matched NC). The delineation of the MML was rated for each bilateral structure (100 GP for NC and 40 GP for PD). We also calculated the average grading scores in each group.

## Statistical Analysis

The average grading scores were expressed as the means ± standard deviation. Scheffe’s analysis of variance (ANOVA) was performed to compare the differences of the average grading scores among PADRE, SWI-like image, and T_2_WI. ANOVA was also performed to compare the differences of average grading scores among H&Y stages. In the comparison of the differences of the average grading scores of each image sequence between age-matched NC and patients with PD we used a Mann-Whitney’s *U* test. We also used a Mann-Whitney’s *U* test to compare the average grading scores between the axial and coronal planes on PADRE images for NC subjects. For the age-matched NC and PD patient groups, chi-squared tests were performed to assess the relationship between the grading scores on both PADRE and SWI-like image and aging or diagnosis of PD. In this test, we compared the proportion of subjects in each grade for the visualization of the MML between two year groups (50–69 vs 70–89 years) and age-matched NC and PD patient groups (non-PD vs PD). A *P* value < 0.05 was considered to indicate a statistically significant difference. The inter-observer agreement for qualitative assessments was calculated as a weighted κ value. The strength of the agreement was considered fair for κ values of 0.21–0.40, moderate for κ values of 0.41–0.60, good for κ values of 0.61–0.80 and excellent for κ values of 0.81 or greater.

## Results

### Delineation of the MML within the GP in NC (Fig. 3A and 3B)

**Fig 3.**
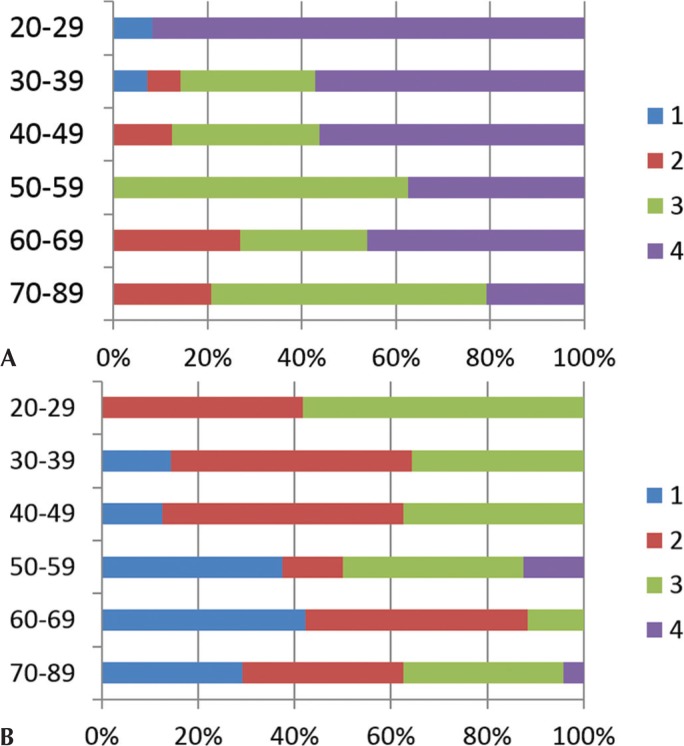
The results of the qualitative assessment in NC (*n* = 50, 100 GP) with PADRE. (**A**) and SWI-like images. (**B**) The distribution of the grading scores according to age demonstrated that most subjects were scored as grade 3 or 4 on PADRE (**A**). The average grading score was 3.31 ± 0.79. Although grade 4 was found in 11 (92%) of 12 GP in 6 younger subjects (less than 30 years old), the proportion of grade 4 decreased and that of grade 3 increased in the elderly subjects equal to or older than 30 years old. Meanwhile less than half subjects were scored as grade 3 or 4 on SWI-like images and the average grading score was 2.11 ± 0.80 (B). PADRE, phase difference-enhanced imaging; SWI, susceptibility-weighted imaging; GP, globus pallidus; NC, normal control subjects.

**Fig 4.**
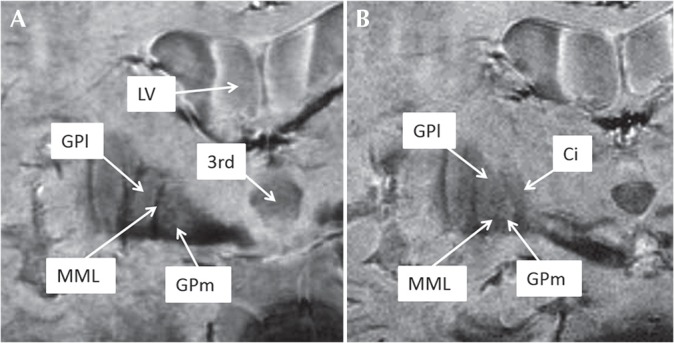
Examples of the grading of the MML on PADRE images from NC, a 30-year-old man, taken on a coronal plane. (**A**) The MML separating the GPm from the GPl can be clearly seen as a thin low signal intensity layer within the GP (grade 4). (**B**) Ci is clearly depicted as a low intensity band, which is adjacent to the GPm anteroposteriorly (posterior slice level as (**A**). GP, globus pallidus; MML, medial medullary lamina; PADRE, phase difference-enhanced imaging; GPm, medial globus pallidus; GPl, lateral globus pallidus; NC, normal control subjects; CI, internal capsule; LV, lateral ventricle; 3rd, third ventricle.

**Fig 5.**
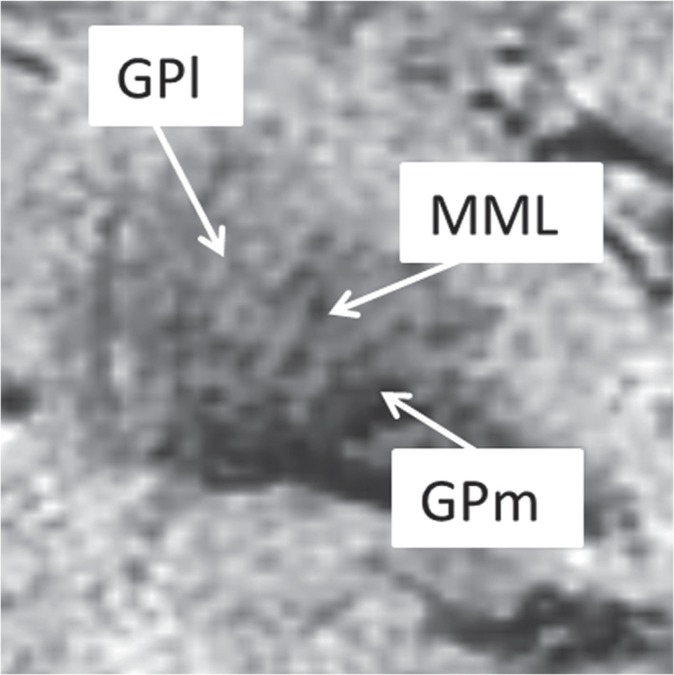
Examples of the grading of the MML on PADRE images from NC, a 70-year-old man, taken on a coronal plane. Although elderly subjects also often showed a good depiction of MML, whole MML was not delineated (grade 3). MML, medial medullary lamina; GPm, medial globus pallidus; PADRE, phase difference-enhanced; GPl, lateral globus pallidus; NC, normal control subjects.

The final consensus scores of grading by two neuroradiologists are summarized in figure 3A and 3B, showing the distribution of the grading scores according to the subject age on PADRE and SWI-like images, respectively. On PADRE images, the depiction of the MML was good (grade 3 or 4) in most cases (84%, 84/100 sides); grade 4 in 49 of 100 sides (49%) and grade 3 in 35 of 100 sides (35%). The average grading score was 3.31 ± 0.79. On PADRE images, grade 4 delineation was noted for nearly all of the subjects younger than 30 years old, in other words, whole MML within GP tended to be identified clearly as a low intensity uninterrupted band on PADRE (Fig. 4A). The proportion of grade 4 decreased and that of grade 3 increased in the elderly subjects equal to or older than 30 years old. Although a part of MML was obscured with age, more than half of MML was still delineated in most elderly subjects (Fig. 5). Although both the coronal and axial planes of PADRE could delineate the internal structure within the GP, the axial planes were superior to coronal for average grading score (3.42 vs 3.09, *P* = 0.019). Meanwhile, the MML was also depicted as a low intensity band on SWI-like images, but the depiction of the MML was better (grade 3 or 4) in less than half cases (34%, 34/100 sides); grade 4 in 2 of 100 sides (2%) and grade 3 in 32 of 100 sides (32%). The average grading score was 2.11 ± 0.80. Most subjects were scored as grade 1 on T_2_WI (the average grading scores was 1.02 ± 0.14). For the average grading score, Scheffe’s ANOVA showed that PADRE image was significantly superior to both SWI-like images (*P* < 0.05) and T_2_WI (*P* < 0.01), and SWI-like images was significantly superior to T_2_WI (*P* < 0.01) in each age group (20–29 years, 30–39 years, 40–49 years, 50–59 years, 60–69 years, and 70–89 years) as well as in whole NC subjects.

For the evaluating PADRE and SWI-like images, the interobserver agreement was good; the κ value was 0.75 and 0.70, respectively. For the evaluating T_2_WI, interobserver agreement was moderate; the κ value was 0.48.

Regarding the visualization of the Ci, the PADRE images were rated as “visible” in all subjects (Fig. 4b), meanwhile the SWI-like images and T_2_WI were rated as “not visible” in all subjects.

### Delineation of the MML in age-matched NC and patients with PD

The depiction of the MML was good (grade 3 or 4) in 33 of 40 sides (83%) for age-matched NC and 36 of 40 sides (90%) for patients with PD on PADRE images. Grade 3 delineation was noted in a large percentage of patients with PD (24/40, 60%) as well as the NC (21/40, 53%). There were no significant differences between the age-matched NC and patients with PD in terms of the average grading score (3.13 ± 0.69 vs 3.20 ± 0.61, respectively).

For the SWI-like images, the best depiction of the MML was rated in 9 of 40 sides (23%) for age-matched NC, and in 17 of 40 sides (43%) for patients with PD. Therefore, the average grading scores for the age-matched NC and patients with PD were 1.90 ± 0.81 and 2.08 ± 1.19, respectively. In both the age-matched NC and patients with PD the depiction of the MML on T_2_WI was grade 1 in all cases. We found no significant difference between age-matched NC and patients with PD for the average grading scores in both SWI-like images and T_2_WI (both, *P* > 0.05).

Moreover, in both age groups (50–69 years and 70–89 years groups), we found no significant difference between age-matched NC and patients with PD for the average grading scores in all image sequences. In both PADRE and SWI-like images, the average grading scores showed no significant relationship with aging and diagnosis of PD (chi-square tests, *P* > 0.05).

For age-matched NC, the interobserver agreement was excellent for PADRE and SWI-like images; the κ value was 0.92 and 0.88, respectively, and good for T_2_WI; the κ value was 0.70. For patients with PD the interobserver agreement was excellent for SWI-like images; the κ value was 0.84, and good for PADRE and T_2_WI; the κ value was 0.66 and 0.69, respectively.

**Fig 6.**
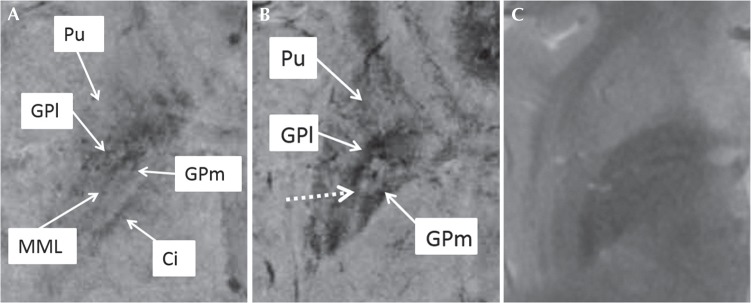
Comparison among PADRE (**A**), SWI-like image (**B**) and T_2_WI (**C**) from a PD patient (a 78-year-old female). The MML can be clearly seen as a thin low signal intensity layer within the GP (grade 4) on PADRE, but not on SWI-like image (grade 1) and T_2_WI (grade 1). The Ci is clearly depicted on only PADRE image. The bright band within the GP in (**B**) (dotted arrows) is assumed an artefact produced during the image-processing steps of SWI-like image. PADRE, phase difference-enhanced imaging; SWI, susceptibility-weighted imaging; WI, weighted imaging. PD, Parkinson’s disease; MML, medial medullary lamina; GP, globus pallidus; Ci, internal capsule; Pu, putamen.

Therefore, PADRE image was significantly superior to the SWI-like images and T_2_WI for the average grading score in both the age-matched NC (3.13 vs. 1.90 and 1.00 respectively) and patients with PD (3.20 vs. 2.08 and 1.00, respectively) (Fig. 6). Regarding the visualization of the Ci, all subjects were rated as “visible” on the PADRE image but not on the SWI-like images and T_2_WI.

For all image sequences, the analyses by ANOVA demonstrated no significant differences of average grading scores among H&Y stages (all, *P* > 0.05).

## Discussion

In neurologically normal subjects there is an increased susceptibility effect in the GP as a result of age-related iron deposition and/or calcification. As MR sequences ( ${\text{T}}_{2}^{*}\text{WI}$ or SWI) are highly sensitive to this effect, this degrades their image quality. Although Nölte et al.^15^ demonstrated that ${\text{T}}_{2}^{*}\text{WI}$ could depict the anatomical structures within the GP, their study subjects were normal young adults and one PD patient. Therefore, studies on more patients with PD and wide range of age NC were needed to evaluate different MRI methods (sequence and orientation) for their visualization of the internal structures within the GP.

Therefore, firstly we investigated the depiction of the MML in a wide range of age NC on PADRE, SWI-like images and T_2_WI, and we demonstrated that on PADRE images the MML was clearly delineated and it was possible to depict the GPm in most NC. In the younger subjects (especially less than 30 years old), the MML tended to be identified as a contiguous low intensity band within GP on PADRE (grade 4) and a part of MML was obscured in elderly subjects with age (grade 3). Some PADRE images yield a new level of contrast that cannot be obtained with older phase techniques. The previous studies suggested that the low signal intensity on PADRE images reflects myelin density rather than iron deposition.^16,17^ Because a part of the MML is derived from nerve fibres of the striatum,^8^ we suppose that the low intensity of the MML on PADRE is due to the presence of myelin. The obscuration of the MML with age may be due to the overall increases in mineralization in the GP and/or a loss of myelin in the MML. Recent investigators reported with quantitative susceptibility mapping (QSM),^24^ which directly reflects the underlying tissue susceptibility, the changes in appearance of MML related to age. In their study, the MML within the GP was delineated in young NC but obscured by about 60 years old. In comparison with the previous study, PADRE could depict the MML even in elderly subjects (over 60 years old). This finding suggests that PADRE may be more sensitive than QSM for depicting myelin.

In both our patients with PD and NC, SWI-like images were inferior to PADRE images for the depiction of the MML within the GP. This may be due to iron deposition and/or calcification in the GP resulting in poor depiction of the MML, thereby limiting the ability to depict the GPm on the SWI-like images. Although the PADRE technique is also designed to enhance phase differences on the magnitude image, it may be less sensitive than SWI to iron deposition or calcification.

Although some previous MR studies which include SWI^25^ or QSM^24^ reported that there were no significant differences in the depiction of iron deposition in the GP between NC and patients with PD no study reported whether the depiction of the MML within GP in patients with PD was different from that in NC. In this study, we found no difference in the depiction of the MML between patients with PD and NC, which may indicate that no pathologic change is occurred in the MML in patients with PD. The current assessment showed no significant differences between the average grading scores on the PADRE images and the H&Y stages. It is important to note that the depiction of the MML was good on the PADRE images even in advanced-stage PD (H&Y stage 3).

Coronal images may be better than axial images to determine the accurate placement of electrodes into the GPm in patients subjected to DBS. In this study, both the coronal and axial planes of PADRE could delineate the internal structure within the GP. Furthermore, it is important to evaluate the relationship between the GPm and the Ci, because the Ci is a vital structure close to the treatment field of DBS. In this study, we found the Ci including the corticospinal tract, which may be difficult to visualize on SWI-like images and conventional T_2_WI, was well delineated on the PADRE images. We also found, on the other hand, that the axial planes were superior to coronal for average grading score (3.42 vs 3.09, *P* = 0.043). One possible reason for this may be due to anatomical locations of the GPm and the GPl. The boundary between the GPm and the GPl is oriented perpendicularly to the axial plane of section and might be shown well on PADRE images. On the other hand, on the coronal-plane images, a part of the boundary may be oriented obliquely to the plane of section, which may provide poor contrast between the GPm and the GPl.

Our study has some limitations. First, the number of MR images was small, especially the number of the patients with PD. Moreover, we did not subject the patients with PD who would undergo the DBS procedure in fact. Further study will be needed including more patients with PD who will undergo the DBS procedure actually to reconfirm the result of this study. Second, although we defined the thin layer of low signal intensity within the GP as the MML on the basis of the cadaveric specimen and a valuable atlas,^23^ we could not correlate our findings with pathological and radiological studies. It is also not known whether and how pathological changes such as iron deposition and/or calcification within the GP are specifically related to the obscuration of the internal structure of the GP. Third, we did not evaluate the geometric accuracy for targeting based on PADRE images. The distortion on PADRE images must be assessed before DBS is undertaken in the clinical setting. Fourth, the SWI-like image that was performed in the present study differed from conventional SWI and could have influenced the results. Lastly, a decrease in the image quality should be taken into account due to the use of head frames and motion artifacts in patients with PD.

In conclusion, the PADRE technique can depict the internal structures of the GP in NC and patients with PD because it delineates the MML, which has been difficult to delineate on conventional MR images.
